# Long-term, low-dose macrolide antibiotic treatment in pediatric chronic airway diseases

**DOI:** 10.1038/s41390-021-01613-4

**Published:** 2021-06-12

**Authors:** Jialiang Sun, Yanan Li

**Affiliations:** Department of Pediatrics, The First Hospital of Jilin University, Changchun, China

## Abstract

**Abstract:**

Macrolide antibiotics are one of the most commonly used broad-spectrum antibiotics. They have an inhibitory effect on a variety of respiratory pathogens; besides, they have non-anti-infective effects, including anti-inflammatory, regulating airway secretion, immune regulation, and other effects. A growing number of studies have shown that the non-anti-infective effects of macrolides have important and potential value in the treatment of pediatric chronic airway diseases; the therapy was described as “long-term, low-dose usage”; unfortunately, there is no guideline or consensus that applies to children. To better carry out the mechanism and clinical research of non-anti-infective effect and promote its rational use in children, the authors summarize the evidence of the usage of long-term, low-dose macrolide antibiotic therapy (LLMAT) in the treatment of chronic airway diseases in children and the progress in recent years.

**Impact:**

- This review summarizes the evidence (mostly in recent 5 years) of the usage of long-term, low-dose macrolide antibiotic therapy in the treatment of chronic airway diseases.
- The recent studies and guidelines support and enrich the point that long-term, low-dose macrolide antibiotic therapy has potential benefit for children with severe asthma, CF, non-CF bronchiectasis, and BO, which provides clinical references and is of clinical interest.
- Long-term, low-dose macrolide antibiotic therapy has good safety, and no serious events have been reported; however, potential cardiac side effects and macrolide resistance should be clinically noted.

## Introduction

Macrolide antibiotics are one of the most commonly used broad-spectrum antibiotics. They also have anti-inflammatory properties, regulating airway secretion, immune regulation, corticosteroid (CS) saving, and other effects. As early as the late 1950s, macrolide antibiotics were used in the treatment of asthma to reduce CS side effects.^1^ In the early 1980s, treatment with macrolide antibiotics was proven to improve the 10-year survival rate of patients with diffuse panbronchiolitis;^2^ this encouraging result has aroused scientists’ attention to the non-anti-infective effects of macrolide antibiotics and attempts to use macrolide antibiotics to treat chronic airway diseases in adults and children.^3,4^ The non-anti-infective usage is to use immune regulation mechanisms other than its direct antibacterial mechanism, such as anti-inflammatory, blocking toxic factors, and inhibiting the formation of biofilms;^5,6^ however, the corresponding research has not yet reached a consensus. Until April 2020, the British Thoracic Society issued guidelines for the application of long-term macrolide antibiotics for adult respiratory diseases and described the use of macrolide antibiotics for immunomodulation as “long-term, low-dose usage,” and it provides specific guidance and recommendations for the treatment of adult asthma, bronchiectasis, chronic obstructive pulmonary disease (COPD), bronchiolitis obliterans (BO), and other respiratory diseases; unfortunately, this guideline does not apply to children.^7^

Therefore, it is urgent to investigate the field with respect to the non-anti-infective effect of macrolide antibiotics in children. Chronic airway diseases in children include chronic rhinosinusitis (CRS), bronchial asthma, cystic fibrosis (CF), BO, etc. The above diseases are all related to one or more of the aspects involving respiratory tract infection and/or injury, chronic inflammation of the respiratory tract, mucus hypersecretion, damage to the mucociliary clearance system, and abnormal epithelial regeneration of the body, and these aspects could interact as causes and effects and vicious circle, causing the diseases protracted.^5,8^ The ideal long-term and low-dose macrolides are drugs with small adverse reactions, a long half-life, and little effect on other combined drugs. Cytochrome P450 (CYP450) oxidase is a superfamily of heme, which is involved in the metabolism of most drugs. CYP450 3A4 enzyme (CYP3A4) is the most common one of the CYP450 family.^9^ Azithromycin has a long half-life, which makes weekly administration possible. Compared with other macrolide drugs, azithromycin does not affect the activity of CYP3A4^9,10^ and has become a research hotspot.^11,12^ In recent years, the evidence-based medical evidence of the anti-inflammatory effects of macrolide antibiotic therapy to treat chronic airway inflammation in children has gradually increased. For example, macrolides represented by azithromycin were used to reduce airway inflammation in children with severe asthma, CF, non-CF bronchiectasis, etc.^5,13,14^ Therefore, this review aims to summarize the evidence of the usage of long-term, low-dose macrolide antibiotic therapy (LLMAT) in the treatment of chronic airway diseases in children and the progress in recent years and provide clinical references.

### The immunomodulatory effect of macrolide antibiotics

Macrolide antibiotics have immunomodulatory effects. In chronic airway diseases, infective or non-infective factors can trigger the release of cytokines, chemokines, and inflammatory mediators by affecting cell signal transduction, producing inflammatory responses mediated by neutrophils, eosinophils, and macrophages.^15^ The anti-inflammatory effects of macrolide antibiotics are performed via regulating cell signal transduction and inhibiting the production of inflammatory cells, cytokines, and inflammatory mediators, thereby playing a role in regulating immunity, as shown in Table 1. For example, macrolide antibiotics could regulate nuclear factor-kB (NF-kB) expression, inhibit the binding activity between NF-kB and DNA, reduce tumor necrosis factor (TNF)-α mRNA and its protein expression levels, prevent neutrophil recruitment into the lung,^16,17^ and interfere with the proliferation of fibroblasts and vascular endothelial cells,^18^ thereby regulating the fibrous tissue and angiogenesis of chronic airway inflammation and improving airway remodeling.^19,20^ In addition, they can also reduce the production of pro-inflammatory cytokines such as interleukin (IL)-1β, IL-6, TNF-α, IL-8, and IL-17,^21,22^ reduce the infiltration of neutrophils and neutrophil elastin in the lung, and inhibit the release of eosinophil-activating chemokines and IL-5 to prevent the recruitment of eosinophils,^23^ thereby reducing cellular inflammatory mediators and superoxide release and protecting the airway epithelial barrier and cilia function.

**Table 1:** Immunomodulatory effects of macrolides on chronic airway diseases.

Effects	Mechanism	Targets	Action
Anti-inflammation	Cell signaling regulation	Interference in the mitogen-activated protein kinase (MAPK) system	Reduce inflammation gene expression and regulate cell growth differentiation
		Modulating the expression of transcription factors (especially NF-kB)	
	Interfere with inflammatory cells and cytokines	Decrease in TGF-β1, PDGF, VEGF	Improve chronic airway inflammation and airway remodeling
		Interference with the proliferation of fibroblasts and vascular endothelial cells	
		Prevention in the recruitment of eosinophils via inhibition of eosinophil-activated chemokines and IL-5 release	
		Improve Th2 inflammation via decrease in IL-4 and IL-13	
		Prevention in the recruitment of neutrophils via inhibition of IL-8 and TNF-α	
Airway secretion regulation	Cell signaling regulation	Interference with intracellular calcium and chloridion signaling pathway	Decrease in airway water and mucus secretion
		Partial activation of the muscarine receptor	
	Interfere with inflammatory cells and cytokines	Inhibition of submucosal mucous adenocytes (goblet cell) via decrease in TNF-α and IL-13	
Immunomodulatory-related antimicrobial effects	Inhibition of microbial adhesion	Inhibition of plant hemagglutinin, hemolysin, etc.	Antimicrobial effect below MIC
	Inhibition of virulence factors	Inhibition of exotoxin A, elastase, etc.	
	Biofilm inhibition	Inhibition of alginate synthesis in biofilms	
	Impair the quorum-sensing system	Blocking expression of signals known as “autoinducers”	

Airway mucus hypersecretion is an important feature of many chronic airway diseases.^5^ Vivo studies and vitro studies have confirmed that macrolide antibiotics can inhibit excessive mucus secretion but do not affect the normal physiological mucus secretion. For example, clarithromycin has been proved to reduce sputum production in lower respiratory diseases, such as chronic bronchitis and bronchiectasis.^24^ The mechanism might be that macrolides affect the chloride ion channels in the airway, partially activate muscarinic receptors, inhibit calcium ion influx in submucosal mucous gland cells, and affect ion channels to reduce airway water and mucus secretion.^25,26^ Some pro-inflammatory cytokines such as TNF-α can stimulate airway goblet cell proliferation and mucus gene expression, while IL-13 can induce goblet cell proliferation and excessive mucus secretion. Macrolide may inhibit mucus hypersecretion by inhibiting the expression of these cytokines.^26^

Macrolide antibiotics can interfere with bacterial protein synthesis even if they are below minimal inhibitory concentration (MIC), and they also have such antimicrobial activity to *Pseudomonas aeruginosa* (PA), which is resistant to macrolides,^27^ indicating that the antimicrobial effect is not dependent on its direct antibacterial activity but is related to its immunomodulatory effect.^28,29^ The immunomodulatory mechanisms of macrolides include inhibition of pathogenic microorganisms from adhering to epithelial cells, blocking of toxic factors to reduce bacterial virulence, and inhibition of microbial biofilm formation in chronic airway infection. The inhibition of PA biofilm formation is of particular concern;^30^ the mechanism may be that macrolide antibiotics inhibit the expression of flagellin to weaken the motility of pseudomonas and inhibit the production of alginate, which is an important component in biofilm formation, thereby inhibiting PA from damaging epithelial cells or stimulating cytotoxicity of neutrophils.^30,31^ Besides, macrolides impair the quorum-sensing system by blocking the expression of signals known as “autoinducers.”^32^

Therefore, the immunomodulatory activity of macrolide antibiotics includes the regulation of immunity at gene, molecule, and cell levels, manifesting as the long-term inhibition of inflammatory response caused by excessive immune cells and inflammatory factors, which is conducive to the reduction of inflammation and airway damage caused by chronic airway diseases.^7,33,34^

### Macrolide antibiotics and chronic airway disease in children

#### Severe asthma

Bronchial asthma (referred to as asthma) is the most common chronic airway disease in childhood characterized by chronic inflammation, bronchial hyperresponsiveness (BHR), and airflow limitation. Most children could be well controlled by standardized treatment, whereas some are still not effectively controlled under standardized treatment; these children have brought a heavy mental and economic burden to the family and society due to the treatment difficulties.^35,36^ Type 2 inflammation is found in nearly 50% of people with severe asthma. It is characterized by excessive helper T cell type 2 (Th2) cell (eosinophils, basophils, and mast cells) activation by secreting IL-4, IL-5, and IL-13. Type 2 inflammation is often characterized by eosinophilia or increased fractional exhaled nitric oxide (FENO) value and may be accompanied by atopy, whereas non-type 2 inflammation is often characterized by increased neutrophils.^37–40^ In mild-to-moderate asthma, the standardized and correct use of inhaled corticosteroid (ICS) can rapidly improve type 2 inflammation, but in severe asthma, type 2 inflammation may be relatively refractory to high-dose ICS; oral corticosteroid (OCS) may be effective, however, the side effects of long-term use mean that alternative treatments should be sought. In recent years, macrolide antibiotics represented by azithromycin have been considered to have the function of regulating and rebuilding Th1/Th2 balance, which is beneficial to patients suffering from severe asthma.^41–43^ In addition, studies have also found that macrolides can antagonize airway remodeling and reduce BHR in recent years,^20,44^ making them a candidate medicine for the treatment of severe asthma.

In the current research on the long-term treatment of severe asthma with macrolides, the results of pediatric studies are encouraging. A meta-analysis showed that, in children with OCS-dependent asthma, macrolide antibiotics (e.g., roxithromycin, 150 mg/day, 8 weeks: troleandomycin, 250 mg twice daily, tapered to 250 mg once daily, 16–136 weeks) could improve pulmonary function forced expiratory volume in 1 s (FEV1) and reduce the daily dose of OCS needed to control the symptom. The degree of dose reduction is directly related to the duration of macrolide therapy. However, long-term treatment with macrolides in asthma patients yields controversial results; a Cochrane review published in 2015 indicated that macrolides therapy did not show advantages over placebo for most clinical outcomes despite that they might be beneficial on some measures of pulmonary function and symptom scales.^45^ Nonetheless, an important limitation is that the relatively small sample size of the studies to date does not allow for a definitive assessment of the effectiveness of macrolides in the treatment of chronic airway inflammation in severe asthma patients.^45,46^ Subsequently, in a randomized controlled trial in pediatrics published in 2016, Wan et al. found that after 4 weeks of clarithromycin treatment at 5 mg/kg, FEV1 was significantly improved, and the FENO value was significantly reduced.^47^ It is noted that azithromycin is the most commonly used macrolide antibiotic in current research.^5^ In most pediatric studies, (azithromycin, 10 mg/kg, consecutive 3 days/weeks, >8 weeks) therapy can significantly improve the FEV1 of children with severe asthma, reduce the risk of repeated wheezing, and reduce the application of rescue drugs. It is found in adult studies that long-term, low-dose use of azithromycin (250 mg thrice weekly, >8weeks) would reduce the exacerbations of non-acidophilic severe asthma^48^ and improve pulmonary function including FEV1 and peak expiratory flow in Caucasians and Asians.^49^ However, there is no consensus on the optimal dose and duration of macrolide antibiotics to produce potential positive effects.^50^ The guideline in adults recommended that if gastrointestinal side effects occur at the higher dose of azithromycin (500 mg thrice weekly) a dose reduction to azithromycin 250 mg thrice weekly could be considered if macrolide therapy has been of clinical benefit;^7^ whether this recommendation applies to pediatric patients deserves further study.

Based on the current evidence, the Global Strategy for Asthma Management and Prevention in 2019 and 2020 (GINA 2019 and GINA 2020) recommends that patients with severe asthma can choose to add low-dose azithromycin therapy (250 mg for 5 days, 3 times a week for 26 weeks) as an add-on treatment;^40,51^ unfortunately, there is no pediatric recommendation. Adult asthma may originate in childhood, and a large proportion of children with asthma lose symptoms in school age and adolescence, while some children have persistent asthma and impaired pulmonary function. They have an increased risk of fixed airflow obstruction and possible COPD in early adulthood, and some adults develop persistent, recurrent, and emerging asthma symptoms after active smoking.^52^ As a result, asthma symptoms may be relieved from childhood to adulthood in some patients, whereas it recurs later in age, and asthma may be truly cured in others. BHR and decreased pulmonary function are also important predictors of persistent symptoms from childhood to adulthood.^53^ Therefore, it is particularly important to improve BHR and pulmonary function in children with asthma in childhood and adolescence.

In brief, a growing body of evidence suggests that azithromycin has a relatively positive effect on improving pulmonary function, BHR, and CS saving, and LLMAT can be a considerable therapy for children with severe asthma.

#### Cystic fibrosis

CF is a common, life-threatening inherited disease that affects about 1 in every 4000 babies born in the United States and is more common in some European countries.^54^ CF is also a common cause of chronic bronchitis and bronchiectasis in children. Patients with CF have impaired mucociliary clearance and are accompanied by chronic and intense inflammation and persistent respiratory infections, such as PA.^55^ Macrolides with a concentration below MIC can destroy the biofilm and reduce the living bacteria number of PA in the biofilm.^27^

Azithromycin is the most commonly used macrolide antibiotic in CF patients. Its therapeutic trials in CF patients and subsequent randomized controlled clinical trials of azithromycin and placebo clearly showed that long-term, low-dose application to >6 months (azithromycin, 5 mg/kg·day) could improve FEV1, increase weight, and reduce the risk of acute pulmonary exacerbation (PEx) in children with CF, etc.^56–61^ FEV1 is an established marker of CF disease progression that is used to capture clinical course and evaluate therapeutic efficacy.^60,62^ Therefore, reducing the decline rate of FEV1 is also an important goal related to reducing the ultimate morbidity or mortality of CF. In a retrospective analysis published in 2020, Nichols et al.^13^ found that, in addition to improving FEV1, the rate of decline of FEV1 in long-term azithromycin patients was 37% lower than that in the control group during the 3 years; however, the beneficial effect on PEx reported in other clinical trials was not seen in their study.

PA is the most common pathogen in the airways of CF patients, and the infection rate increases with age.^63^ Intravenous and nebulized tobramycin is commonly used to treat PEx in patients with CF. The potential interactions between the mechanisms of antibiotic resistance in the current CF treatment regimen remain unclear, and it is also one of the issues to be resolved in the current CF treatment consensus.^64^ Azithromycin was found to have an antagonistic effect against tobramycin in some PA strains in vitro studies.^65^ There are also cohort studies showing that long-term oral administration of azithromycin may reduce the efficacy of atomized inhalation of tobramycin in adults,^63^ which should be used after weighing the advantages and disadvantages.^13,66^ A retrospective study published in 2020 concluded that azithromycin did not affect the efficacy of inhaled tobramycin or inhaled amtrinanlysine.^13^ There is no evidence currently which shows that azithromycin antagonizes other antibiotics used for the treatment of CF in children, the beneficial results of azithromycin in pediatric CF may not apply to adults, and more high-quality randomized controlled trials are needed. In conclusion, current evidence suggests that macrolides, represented by azithromycin, may have a positive benefit for PEx in pediatric CF patients,^13,58,66^ and long-term azithromycin treatment may be beneficial for children with non-PA infection.^67^

#### Non-CF bronchiectasis

Bronchiectasis is an important chronic airway disease harmful to children’s health, which has a certain disability and fatality rate, resulting in a heavy social burden. Primary immunodeficiency disease, ciliary dyskinesia, infection, and congenital malformation are common causes of non-CF bronchiectasis.^68^ In non-CF bronchiectasis, bacterial colonization and repeated infection can cause the production of a variety of pro-inflammatory mediators (such as IL-6 and IL-8), lead to neutrophil recruitment and elastase release, enhance the release of pro-inflammatory cytokines, which damage the airway ciliated epithelium, cause airway secretions increase, increase airway obstruction, airway poor drainage, and worsen the infection.^69^ Macrolides have been proved to improve airway mucus hypersecretion and chronic airway inflammation in patients with CF due to their immunomodulatory properties.^24^ Therefore, more and more attention has been paid to the therapeutic effect of CF and non-CF patients.^4^

Macrolide antibiotics were found to improve BHR in children.^70^ In recent studies, Gao et al.^71^ analyzed data from 9 studies (559 patients, 3 of which were for children) showing that the results in children and adolescents were similar to those in adults: the use of macrolides not only reduced the acute exacerbation of non-CF bronchiectasis and improved FEV1, sputum volume, and clinical scores but also increased the risk of diarrhea and bacterial resistance. A subsequent meta-analysis showed that LLMAT effectively reduced the progression of bronchiectasis and hospitalization for infectious deterioration in both adults and children with non-CF bronchiectasis, especially for patients with frequent exacerbations and hospitalization, which was different from that of Gao et al.^71^ LLMAT does not increase the risk of adverse events, while it may increase the risk of macrolide resistance.^69^

In conclusion, there are no serious adverse events associated with long-term administration of macrolide antibiotics, and the longest period of application in pediatric studies is 52 weeks (Erythromycin, <15 kg 125 mg, >15 kg 250 mg per day).^67^ However, the type, dose, and course of treatment of macrolide antibiotics used in evidence-based evidence are different at present, and the problem of drug resistance caused by long-term use should not be ignored.^67,72^ The current evidence is not strong enough to recommend macrolides therapy for all non-CF bronchiectasis patients. Therefore, the dose–response relationship between macrolide antibiotics and benefits needs to be further studied.

#### Bronchiolitis obliterans

BO is a pathological concept, which refers to the injury of bronchiolar epithelium and subepithelial tissue due to inflammation and immune response, leading to pathological changes due to the imbalance of the tissue repair process.^73^ Kavaliunaite et al.^74^ classified BO into three categories: post-infectious BO (PIBO), BO post lung transplantation (LT), and BO after bone marrow transplantation (BMT) or hematopoietic stem cell transplantation (HSCT). The pathogenesis of BO is mainly related to the elevation of angiogenesis and key mediators of fibrogenesis such as transforming growth factor-β1, platelet-derived growth factor, and vascular endothelial growth factor or the development of chronic allograft dysfunction. These factors are known to play a role in airway inflammation and remodeling.^18,75^ Respiratory epithelial repair disorders can lead to pulmonary fibrosis and airway remodeling, which is currently believed to be associated with asthma and COPD. The recent research also shows that the disorder of small airway epithelial repair is also involved in the pathogenesis of BO after LT.^76^ Vitro studies have shown that long-term use of macrolides can inhibit vascular endothelial cell migration and angiogenesis, thereby improving airway remodeling and progression of BO.^44,76–80^

Infection is the first cause of BO in children, and the most common pathogen of PIBO is adenovirus (especially serotypes 3, 7, and 21). A retrospective analysis conducted by Chan et al.^81^ showed that children with PIBO were generally non-progressive, some of them could be clinically improved, and the outcomes of BO after transplantation were usually worse. Compared with other post-transplant BO, PIBO tends to have no progress and has a relatively low mortality rate,^82^ which may be due to the continuous growth and development of children and the compensatory effect of healthy lungs. At present, there is no controlled intervention study on long-term treatment of PIBO with macrolide antibiotics. In a prospective study published in 2014, the authors reported a long-term combination of low-dose oral azithromycin (5 mg/kg per day, consecutive 3 days/week, 6 months) and CSs in the treatment of PIBO, 84% of the children achieved useful results in pulmonary function and imaging findings, but the specific role of azithromycin in the therapy is indefinite because there is no control group and the sample size is small.^83^ Therefore, there is no evidence currently to confirm the efficacy of long-term azithromycin in the treatment of children with PIBO.

Patients who received LT, BMT, or HSCT were more likely to develop BO over time after transplantation.^84^ Available evidence suggests that azithromycin can benefit many post-transplant BO patients, including improving FEV1 and reducing airway neutrophils and IL-8 levels.^85–87^ In recent years, azithromycin has achieved many encouraging results in the long-term treatment of BO after LT; the evidence mainly comes from adult studies because the overall number of children’s LT is limited.^19,76^ A retrospective study^86^ found that prophylactic long-term, low-dose oral azithromycin (250 mg/d, 3 times/week, 2 years) improved freedom from BO syndrome 2 years after LT, including improving pulmonary function (forced vital capacity, FEV1, and FEV1%) and functional exercise ability. A recent study found that long-term use of azithromycin (250 mg) could improve pulmonary function and slow down the progression of BO after LT.^88^ As for BMT and HSCT, early studies have shown that macrolides can improve pulmonary function and disease stability in patients with BO after allogeneic BMT.^89^ However, compared with BO after LT, the effect of macrolides in preventing BO after allogeneic BMT/HSCT does not seem to be optimistic. A retrospective study by Jo et al.^90^ in 2015 found that prophylactic azithromycin did not seem to prevent the development of BO after BMT;^90^ in a subsequent randomized, double-blind placebo-controlled trial, Bergeron et al.^91^ found that early administration of azithromycin led to worse airflow decline-free survival than placebo, which also led to the discontinuation of early trials. Generally speaking, azithromycin therapy on BO patients is more popular, but its long-term impact on the quality of life and prognosis seems to be quite different in patients after LT and BMT/HSCT. The current evidence is not strong enough to justify the routine use of macrolides in BO treatment; however, some categories of BO patients, such as children with PIBO and BO after LT, may benefit from macrolide effects. Therefore, the specific beneficiary population, mechanism, and usage of LLMAT in post-transplant BO patients need to be further studied.

#### Chronic rhinosinusitis

CRS is a common chronic airway disease in pediatrics.^92^ The treatment includes short-term anti-infective therapy, intranasal steroid therapy, nasal irrigation, and endoscopic sinus surgery, but there are still 20% of patients whose symptoms are difficult to control after standard internal and surgical treatment, which is called recalcitrant CRS.^93^ At present, it is believed that CRS is a group of heterogeneous diseases caused by the interaction of multiple factors, and its etiology and pathogenesis are not completely clear. Infection, abnormal anatomical structure, abnormal ciliary movement, chronic inflammation, tissue remodeling, systemic and environmental factors, etc., alone or interaction may lead to the persistence of CRS.^93,94^ Bacterial biofilm formation and superantigen formation are important mechanisms for evading host immune response and could be important factors for the persistence of recurrent and recalcitrant CRS inflammation.^95,96^ Besides, CRS is a disease in which local tissue inflammation is strongly biased toward Th2 inflammation. Therefore, medicine to prevent the formation of bacterial biofilm and inhibit Th2 inflammation has become one of the options for the treatment of recalcitrant CRS.

Based on the macrolide effects, researchers have successively used macrolides in the treatment of CRS. An early study published in 2003 showed that clarithromycin [5–8 mg/(kg·day), 8–15 weeks] have a certain therapeutic significance for pediatric CRS^97^; however, due to the lack of placebo control, this study has some limitations. Regrettably, there have been no large-sample, randomized, placebo-controlled clinical trials targeting children. A meta-analysis from 2018 showed that LLMAT can significantly improve nasal endoscopy and computed tomography scores in adult CRS patients, clarithromycin 500 mg BID for 2 weeks and 250 mg QD for 6 weeks^98^ and azithromycin 250 mg QD for 12 weeks^3^ could be used as a reference. LLMAT can theoretically relieve symptoms and improve the endoscopic performance of CRS; however, the current clinical evidence is susceptible to random biases due to lack of power, and it is still not clear about the indications and prognostic effects of long-term macrolide therapy in children with CRS based on current evidence. Therefore, it is necessary to conduct large sample randomized controlled trials to provide evidence. Routine macrolide therapy is not recommended by the latest guidelines for children with CRS,^7,92^ but based on theoretical benefits, it could be considered as an alternative therapy after weighing the advantages and disadvantages in the children with recalcitrant CRS.

### Safety of long-term, low-dose macrolide antibiotic therapy

The potential harm of long-term use of antibiotics is concerned. The effect of macrolides on drug resistance of common respiratory pathogens is an important factor to be considered in long-term use. Current evidence suggested an increased risk of macrolide-resistant *Streptococcus pneumoniae*, *Staphylococcus aureus*, *Haemophilus influenzae*, etc., but did not increase the risk of respiratory exacerbations, intravenous antibiotics, or hospitalization.^67,72^ The effects of macrolides on respiratory pathogens and the optimal duration of efficacy of macrolide therapy need to be further studied. As for side effects, studies showed that children and adults treated with macrolides for a long time had a significantly higher incidence of diarrhea, no significant difference in nausea or vomiting compared with the control group, and had nothing to do with the type of macrolides.^67,71,99^ Moreover, the treatment was well tolerated and there were no patient drop out of the study because of diarrhea.^67,71,99^ The tolerance of azithromycin is better than that of other macrolides. Current data show that LLMAT in pediatric patients is well tolerated and no serious cardiovascular events have been reported. However, since there is no official recommendation for electrocardiogram examination before children take macrolide antibiotics, it is necessary to note potential cardiac side effects such as QT interval prolongation reported in adults.^100^ Therefore, long-term macrolide antibiotics therapy has good safety in children, the continuous evaluation of heart safety in the course of use cannot be ignored, and diarrhea should not be excessively worried. In conclusion, the clinical dilemma is that optimal dose and duration of macrolide antibiotics to produce positive effects in pediatric chronic airway diseases are still indefinite, and it is important to further evaluate the treatment options and advantages and disadvantages of LLMAT.

## Conclusion

Macrolide antibiotics not only have a bacteriostatic effect but also have a certain immunomodulatory effect. Compared with other immunomodulators, macrolide antibiotics have the advantages of fewer adverse reactions and convenient application. For the pediatric chronic airway diseases described in this review, macrolide antibiotics, especially azithromycin, have shown beneficial effects in recent years. It can improve the pulmonary function of children with severe asthma, CF, non-CF bronchiectasis, and BO; reduce the exacerbations of CF and non-CF bronchiectasis; and may improve the prognosis of BO after LT; however, the current clinical evidence is not strong enough to recommend macrolide therapy as the first choice due to the lack of high-quality, multi-center clinical studies. Besides, there is still a lack of clinical evidence for the macrolide effectiveness of PIBO, BO after BMT/HSCT, and CRS in children. Nonetheless, macrolide antibiotics’ long-term use has begun to enter the treatment of chronic airway diseases in recent years as a promising therapy, which has been proved potentially beneficial. Therefore, it is extremely important to further evaluate the treatment options and advantages and disadvantages of macrolides in children with chronic airway inflammation, which is also helpful to form guidelines for the macrolide antibiotic therapy of pediatric diseases, like the guidelines for adults,^7^ and provide references for clinical practice in pediatrics.
